# High Value‐Added Secondary Raw Material From Winemaking Waste Bentonite to Design Antioxidant Pectin‐Based Mucoadhesive Buccal Films

**DOI:** 10.1002/open.70236

**Published:** 2026-05-29

**Authors:** Giulia Di Prima, Cecilia La Mantia, Viviana De Caro

**Affiliations:** ^1^ Dipartimento di Scienze e Tecnologie Biologiche Chimiche e Farmaceutiche (STEBICEF) University of Palermo Palermo Italy; ^2^ Dipartimento di Medicina di Precisione in Area Medica Chirurgica e Critica (MePreCC) University of Palermo Palermo Italy; ^3^ Centro interdipartimentale di Riutilizzo bio‐based degli scarti da matrici agroalimentari (RIVIVE) University of Palermo Palermo Italy

**Keywords:** antioxidants, drug delivery, materials science, sustainable chemistry, thin films

## Abstract

Buccal films are versatile formulations providing targeted drug delivery while ensuring patient comfort, easy administration, and improved efficacy. Additionally, they act as wound dressings, making them suitable to treat painful oral lesions associated with conditions such as oral mucositis, oral lichen planus, and recurrent aphthous stomatitis. Considering the oxidative stress‐related nature of these disorders, natural antioxidants as polyphenols represent promising candidates for locoregional therapy. The aims of this work were to enhance polyphenols recovery from waste bentonite, a wine industry by‐product, to obtain a sustainable high value‐added secondary raw material, and to directly exploit it for buccal film formulation. Polyphenols recovery was maximized through a multiple extraction process using PEG200 as solvent. By investigating both dry and wet matrices and different agitation methods, the impact of water in the original waste and the importance of waste solvent mixing were highlighted. The best balance between extraction efficiency and cost‐effectiveness was identified and the selected extract, named Bentophen200, was used to produce buccal films based on citrus pectin alone (BentoPect) or combined with hyaluronic acid (BentoPect‐Hyalu) by green solvent casting. Showing lower swelling, stronger mucoadhesion, and greater mucosal polyphenols accumulation, BentoPect emerged as potential therapeutic agent for prolonged mucosal contact and pain relief.

## Graphical abstract

Hyaluronic acid and/or pectin (crosslinked by calcium lactate) allowed the development of Bentophen200 extract‐loaded buccal films. Given i) the antioxidant power of the polyphenols‐rich extract optimized via multiple maceration of waste bentonite and ii) the flexibility, mucoadhesivity, and suitability of the films for oromucosal application, they can be considered promising candidates for the prevention and treatment of oral mucositis, oral lichen planus, and recurrent aphthous stomatitis lesions.

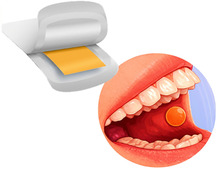

## 1 Introduction

Mucoadhesive buccal films represent efficient dosage forms for oromucosal drug delivery due to their versatility, ease of application, flexibility, small size, and the comfort they provide to patients [1]. These characteristics allow simple administration and support the development of personalized therapeutic strategies, for instance, by modulating the size of the applied film [2, 3]. From a technological perspective, mucoadhesive buccal films are thin and flexible systems designed for the controlled release of actives through the buccal mucosa. The use of mucoadhesive polymers ensures strong adhesion to the mucosal surface and provides adequate mechanical properties, while the addition of plasticizers, such as glycerol, propylene glycol, and polyethylene glycol, improves flexibility and ductility, enhancing the conformability of the film to the mucosa and the overall acceptability of the formulation [3]. Additionally, one of the main advantages of these systems is their potential wound dressing function. Indeed, buccal films can increase the contact time between the active compound and the site of action while simultaneously protecting the injured mucosa, thereby reducing local irritation and the pain perceived by the patients [4]. Thanks to these features, mucoadhesive buccal films may be used in the treatment of several oromucosal lesions. Among the most relevant oromucosal chronic inflammatory conditions associated with painful ulcerative lesions, there are oral mucositis (OM), oral lichen planus (OLP), and recurrent aphthous stomatitis (RAS) [5, 6, 7].

OM is an acute inflammatory condition of the oral mucosa characterized by painful ulcerations, edema, and erythema, which may also extend to the pharyngeal, laryngeal, and esophageal mucosa [8, 9]. OLP is a chronic immune‐mediated mucocutaneous inflammatory disorder currently considered a T‐cell‐mediated autoimmune disease. Its pathogenesis involves complex interactions between host immune responses, pro‐inflammatory cytokine production, and potentially pathogenic microorganisms that contribute to the persistence of chronic inflammation [6, 10]. Additionally, according to the World Health Organization, OLP is a potentially malignant disorder, as it might lead to oral squamous cell carcinoma [11]. RAS is characterized by the recurrent appearance of painful ulcers within the oral cavity, often in the absence of identifiable systemic or local causes [7]. Local treatment of the mentioned inflammatory and oxidative stress‐related diseases of the oral cavity may represent a particularly advantageous therapeutic approach. Among the molecules of interest, polyphenols are currently under the spotlight. They are natural compounds characterized by low bioavailability when administered orally [12]. Polyphenols are composed by one or more aromatic rings bearing hydroxyl groups [13]. This chemical structure confers strong antioxidant properties, allowing the neutralization of reactive oxygen species and contributing to the reduction of oxidative stress and inflammatory processes, and thus potentially useful in the prevention or adjuvant treatment of the mentioned oxidative stress‐related oral pathologies [14, 15, 16]. As polyphenols are plant‐derived secondary metabolites widely distributed in fruits, their recovery from agrifood by‐products represents a sustainable strategy for their use. In the wine industry, for example, only 30–40% of the phenolic compounds present in grapes are transferred to wine, while a significant fraction remains in processing residues, which thus represent a valuable source of these bioactive molecules [17]. The recovery of these compounds fits within a circular economy framework aimed at waste valorization and promotes their green use in the cosmetic and pharmaceutical sectors [18, 19, 20]. In recent years, research has been mainly focused on recovery of the organic by‐products of the wine industry such as pomace, lees, and grape seeds, which are well known for their high polyphenol content and have been used in the development of innovative cosmetic products [21]. However, some inorganic wastes, such as white and black bentonites, also represent a valuable although scarcely explored resource. Bentonite clays are mainly composed of montmorillonite and are the most used fining agent due to their high deproteinizing power, leading to clear and stable products [22, 23]. However, during the clarification process, they adsorb various compounds present in must or wine, including polyphenols, and once used they cannot be regenerated, thus contributing to the generation of large amounts of waste [24, 25]. Recently, waste bentonites have been proven as source of polyphenols. In particular, in recent years, studies by Di Prima et al. have developed sustainable extraction methods based on green unconventional solvents suitable for cosmetic and pharmaceutical applications. These studies demonstrated the possibility of obtaining high value‐added liquid extracts, which can also be converted into polyphenols‐rich powders, exhibiting antioxidant activity, and directly useful to formulate cosmetics or pharmaceuticals, according to a waste‐to‐market approach [24, 26, 27, 28].

Based on these assumption, this work introduces a multistep green extraction process to enhance the recovery of biophenols from waste black bentonite using polyethylene glycol 200 as green solvent. The biobased extract is combined with citrus pectin affording mucoadhesive buccal films, named BentoPect and BentoPect‐Hyalu, for the local treatment of oromucosal oxidative stress‐related diseases such as OM, OLP, and RAS.

## 2 Experimental Section

### 2.1 Materials

*Trans*‐resveratrol (RSV), sodium hyaluronate (HA), β‐cyclodextrins (β‐CD), and calcium lactate pentahydrate were purchased from A.C.E.F. S.p.A. (Fiorenzuola D’Arda, Italy). Gallic acid (GA), the Bradford reagent, and the Folin–Ciocalteu reagent were obtained from Merck (Darmstadt, Germany). Quercetin (QRC) was obtained from Farmalabor (Canosa di Puglia, Italy). Polyethylene glycol 200 (PEG200), 2,2‐diphenyl‐1‐picrylhydrazyl radical (DPPH), and bovine serum albumin (BSA) were supplied from Carlo Erba (Milan, Italy). Trehalose was obtained from Hayashibara Shoji Inc. (Okayama, Japan). Citrus pectin (degree of esterification: 58.0–62.0%) was purchased from Galeno (Comeana, PO, Italy). The isotonic solution, isotonic solution containing trehalose, simulated saliva pH 6.8, citrate buffer pH 5.5, and citrate buffer pH 5.5 containing β‐CD were prepared according to the literature [29]. All salts, chemicals, and solvents were purchased from Carlo Erba (Milan, Italy) and used without further purification. Porcine buccal mucosa specimens were kindly provided by Azienda Agricola Mulinello S.r.l. (Leonforte, Enna, Italy) and were collected from freshly slaughtered domestic 6–8‐month‐old pigs intended for human consumption (ethical approval unrequired). The waste black bentonite, indicated as BB (Enobent Standard + activated carbon in the 1:1 w/w ratio), was supplied by Bono & Ditta S.p.A. (Campobello di Mazara, Trapani, Italy). Following the clarification of organic white grape must, a sample of the bentonite was frozen at −20°C, transported in refrigerated containers to the research laboratories, pulverized, sieved, aliquoted, and subsequently stored at −80°C. A portion of the obtained waste was subjected to freeze‐drying, thus yielding two distinct waste materials to be further used referred to as BB dry (freeze‐dried) and BB wet (as originally received) [24, 26, 27, 28].

### 2.2 Multiple Maceration Process for Polyphenols Recovery from Waste Black Bentonite

Fifty grams of BB wet or 25 g of BB dry were mixed with 200 g of PEG200 and subjected to maceration under vigorous stirring (magnetic stirring or mechanical stirring) for 1 hr at 25.0  ±  0.5°C, protected from light. Afterward, the resulting homogeneous suspensions were centrifuged for 1 hr at 5500 rpm and the colored supernatants were collected, weighed, pooled, and mixed with a new, accurately weighed aliquot of bentonite to maintain a constant bentonite/solvent weight ratio (1:8 w/w if using BB dry; 1:4 w/w if using BB wet). The extraction procedure was repeated up to a maximum of seven steps. For each extraction step, a small aliquot (3 g) of the extract was withdrawn, filtered (PTFE, 0.22 μm), stored at 4°C protected from light, and used for subsequent characterizations.

Samples from the multiple maceration process were indicated as follows:

• DMa1‐7: extractions performed by using BB dry under magnetic stirring (the number indicates the extraction step);
• WMa1‐7: extractions performed by using BB wet under magnetic stirring (the number indicates the extraction step);
• WMe1‐7: extractions performed by using BB wet under mechanical stirring (the number indicates the extraction step).

For each extraction step, the recovery % was calculated as follows

$$\text{Recovery}\;\%=\frac{\text{Recovered extract }(g)}{\text{Starting amount}\;\mathrm{of}\;\mathrm{PEG}200\;(g)}\times 100$$

Each multiple maceration process was repeated four times and results are reported as mean (*n* = 4) ± standard error (SE). Control samples were obtained by using fresh BB (1:8 w/w bentonite/solvent ratio; magnetic stirring).

### 2.3 Extracts Characterization

The aliquots obtained from each extraction steps were evaluated as previously reported [24, 26, 27, 28] in terms of: i) density (reported as g/mL); ii) pH after water dilution; iii) GA, RSV, and QRC concentration by HPLC–DAD analyses (reported as μg/mL); iv) total phenolic content (TPC) by Folin–Ciocalteu assay (reported as mg_GAE_/g); v) total protein content (TPtC) by Bradford assay (reported as mg_BSA_/g); vi) antioxidant power by DPPH assay (reported as mg_GAE_ _60 min_/g). The evaluations were repeated in triplicate on each extraction and results are reported as mean (*n* = 12) ± SE. Results from the control samples were not reported as no chromatographic peaks were detected neither antioxidant power, phenolic and protein content emerged.

### 2.4 Preformulation Studies: Bentophen200 Stability

Following the aforementioned studies, the WMe‐3 extract was selected for the further preparation of the buccal films and it was indicated as Bentophen200 extract. Its stability was then preliminarily assessed both when stored at 4°C protected from light (storage stability) as well as under controlled temperature and humidity conditions (thermal stability), selected according to the subsequent solvent‐casting drying process employed for the preparation of the buccal films. For the storage stability, the Bentophen200 extract was analyzed monthly until 6 months by Folin–Ciocalteu, Bradford, and DPPH assays as well as through HPLC–DAD analyses. Concerning the thermal stability, 2 g of Bentophen200 extract (*W*_0_) was placed in polystyrene molds with a surface area of 21.23 cm^2^ and subjected to thermal treatment in an oven at 30 ± 1°C in the presence of anhydrous CaCl_2_ (RH: 60%) for 48 h. At the end of the drying process, the extract was analytically weighed (*W*_d_), and the percentage weight loss was calculated using the following equation

$$\text{Weight}\;\;\mathrm{loss}\;\%=\frac{W_{0}-W_{d}}{W_{0}}\times 100$$

The experiment was performed in triplicate, and results are expressed as mean (*n* = 3) ± SE. Taking into account the amount of water lost by evaporation from the extract, HPLC–DAD analysis, Folin–Ciocalteu assay, and DPPH assay were subsequently repeated as previously described. These analyses were carried out in duplicate for each sample, and results are reported as mean (*n* = 6) ± SE.

### 2.5 Preparation of the Bentophen200 Extract‐Loaded Buccal Films

The Bentophen200 extract‐loaded buccal films were prepared using the solvent casting technique, following the preparation of a dispersion as described below. A 3% (w/w) citrus pectin dispersion in ultrapure water was first prepared under constant magnetic stirring at 60.0 ± 0.5°C. Simultaneously, a 1% (w/w) sodium hyaluronate (HA) dispersion and a 5% (w/v) calcium lactate pentahydrate solution were prepared in ultrapure water. Subsequently, appropriate amounts of the citrus pectin dispersion were weighed into a beaker and supplemented with the required quantity of the HA dispersion (when present). The Bentophen200 extract was then added, and the mixture was maintained under constant stirring at room temperature and protected from light for 1 h. The appropriate amount of the calcium lactate pentahydrate solution was then added, and the resulting dispersion was kept under continuous stirring overnight, protected from light. Accurately weighed quantities of the resulting dispersion were then poured into circular polystyrene molds (surface area: 21.23 cm^2^) and allowed to dry in an oven at 30 ± 1°C for 48 h in the presence of anhydrous CaCl_2_ (RH: 60%). The formulation containing only citrus pectin as polymer was indicated as BentoPect, while the one comprising citrus pectin and HA was named BentoPect‐Hyalu. The qualitative and quantitative composition of the dispersions used for the preparation of each film is shown in Table 1.

**TABLE 1:** Quantitative composition (expressed as weight and dry weight) of the components of the dispersion poured into 21.23 cm^2^ molds for the preparation of the BentoPect and BentoPect‐Hyalu buccal films.

	BentoPect		BentoPect‐Hyalu	
	Weight, g	Dry weight, mg	Weight, g	Dry weight, mg
**Bentophen200 extract**	0.936	739.066	0.936	739.066
**Citrus pectin (3% w/w)**	7.905	237.150	6.916	207.501
**HA (1% w/w)**	—	—	2.965	29.650
**Calcium lactate pentahydrate (5% w/v)**	0.159	0.008	0.140	0.007
**Total weight per mold**	9.000	976.224	10.957	976.224

The dried buccal films were removed from the molds and weighed using an analytical balance. Each type of film was prepared in triplicate, and results are expressed as the mean (*n* = 3) ± SE.

### 2.6 Folding Endurance

Each film was repeatedly folded at the same point 300 times, and the procedure was then repeated at different locations. The folding endurance, defined as the maximum number of folds a film can withstand without breaking, was used to assess flexibility. Films that remained intact after 300 folding cycles were considered resistant and flexible [30, 31].

### 2.7 Uniformity Studies: Weight, Thickness, and Extract Content

To evaluate the weight uniformity of the prepared buccal films, four disks (area 0.38 cm^2^) were cut from each film sampling different regions of the same film (two from the center and two from the periphery). The disks were then accurately weighed using an analytical balance with precision to the fifth decimal place. Results are reported as mean (*n* = 12) ± SE.

The thickness uniformity of each buccal film was determined by measuring the thickness at five different points per film using a digital micrometer with a measurement range from 0 to 25 mm and a sensitivity of 0.001 mm. Results are reported as mean (*n* = 15) ± SE.

For the study of content uniformity and the determination of the loading efficacy % (LE%), each previously obtained film disk was placed in an amber volumetric flask and completely dissolved in 5 mL of ultrapure water. The resulting clear solution was then subjected to Folin–Ciocalteu and DPPH assays, as previously reported [24, 26, 27, 28]. The LE% was calculated with respect to the theoretical TPC and antioxidant power, taking into account the amount of extract incorporated into each disk, according to the following equation

$$\mathrm{LE}\%=\frac{{\mathrm{TPC}}_{\mathrm{film}\;\mathrm{disc}}}{{\mathrm{TPC}}_{\text{theoretical}}}\times 100\;o\;\frac{{\text{Antioxidant power}}_{\mathrm{film}\;\mathrm{disc}}}{{\text{Antioxidant power}}_{\text{theoretical}}}\times 100$$

The experiments were repeated twice, and results are reported as mean (*n* = 6) ± SE.

### 2.8 Swelling Studies

The swelling capacity of the buccal films was evaluated by means of gravimetric swelling tests as well as radial and longitudinal swelling assays. Film disks with an area of 0.95 cm^2^ were accurately weighed (*W*_0_), placed on watch glasses and wetted with 500 µL of artificial saliva pH 6.8. At predetermined time intervals (5, 15, 30, 45, and 60 min), any excess fluid was carefully removed using absorbent paper, and the disks were reweighed (W_t_). The swelling index at each time point was then calculated according to the following equation

$$\text{Swelling index}=\frac{W_{0}+(W_{t}-W_{0})}{W_{0}}$$

The experiment was performed in triplicate, and results are expressed as mean (*n* = 3) ± SE. The radial and longitudinal swelling test was carried out by placing a film disk with an area of 0.95 cm^2^ on a microscope slide positioned over graph paper. The sample, wetted with 500 µL of artificial saliva pH 6.8, was observed for a maximum period of 1 h, with photographs taken at predetermined time points to assess the variations in the disk’s diameter and thickness. The experiment was conducted in triplicate, and results are reported as mean (*n* = 3) ± SE.

### 2.9 Mucoadhesion

#### 2.9.1 Qualitative Mucoadhesion Assay

For the qualitative assessment of the mucoadhesive properties of the buccal films, porcine tissue samples were used. These were collected from the vestibular region of the retromolar trigone of animals intended for human consumption, with an average age of 12 months. The tissues were excised immediately after slaughter and transported to the laboratory in refrigerated containers within 2 h. Upon arrival, the samples were rinsed with isotonic solution and, after removal of excess connective and adipose tissue, immersed in an isotonic solution containing 5% (w/v) trehalose for ≈1 h. They were then stored at −80°C until use. For the mucoadhesion test, the tissue samples were thawed and equilibrated in prewarmed (37.0 ± 0.5°C) artificial saliva pH 6.8 for about 10 min. The tissue was then placed on a Petri dish, and a film disk with an area of 0.95 cm^2^ was applied onto its surface. Following a brief pressure applied for 5 s by placing a 20 g weight on top of the film, the tissue samples were subjected to movements simulating normal oral cavity actions as well as the effect of gravity. The experiment was performed in duplicate for each film, and both photographs and videos were recorded during the procedure.

#### 2.9.2 Quantitative Mucoadhesion Studies

For the quantitative assessment of the mucoadhesive properties of the buccal films, porcine buccal mucosa was recovered from the porcine buccal tissues stored at −80°C. The latter were thawed and subjected to thermal shock by immersion for 1 min in isotonic solution at 60.0 ± 0.5°C, in order to separate the mucosal epithelium from the underlying connective and adipose tissues. The buccal mucosa was then manually detached [32]. For the mucoadhesion studies, a Texture Analyzer TX‐700 equipped with a 10 N load cell (Lamy Rheology, Champagne‐au‐Mont‐d’Or, France) was used. The instrument was fitted with a cylindrical probe (Model TX‐BLMPG; diameter: 12.7 mm, height: 30 mm; contact area: 1.26 cm^2^) made of polymethylmethacrylate (PMMA). Film disks with an area of 1.26 cm^2^ were fixed to the probe using double‐sided adhesive tape, whereas the porcine buccal mucosa was mounted on a custom‐made PMMA support and moistened with artificial saliva pH 6.8. The study was performed as follows: i) in the resting position, the mucosa and the formulation were not in contact; ii) the experiment started when the probe touched the mucosal surface (probe descent speed: 0.2 mm/s) and penetrated it to a compression depth of 1 mm; iii) the probe applied a constant maximum force for a variable contact time (holding position: 1 mm; contact time ranging from 10 to 45 s); iv) the probe then returned to the resting position (withdrawal speed: 0.1 mm/s), recording the force required to detach the mucosal surface from the formulation (detection threshold: 0.04 N) [33].

The results obtained in terms of adhesion force were normalized to the surface area of the formulation, and the detachment force was calculated as follows

$$\text{Detachment force }\left(\frac{N}{m^{2}}\right)=\frac{\text{Force}\;\mathrm{of}\;\text{adhesion }(N)}{\text{Contact}\;\mathrm{area}\;(m^{2})}$$

The experiment was repeated six times and results are reported as mean (*n* = 6) ± SE.

### 2.10 Ex Vivo Permeation and Accumulation Studies

The ex vivo assays were performed by mounting appropriate sections of the porcine buccal mucosa between the donor and acceptor chamber of Franz‐type vertical diffusion cells. Prior to the loading of the buccal films, the Franz diffusion cell—whose donor compartment had been filled with citrate buffer pH 5.5 and acceptor chamber with citrate buffer pH 5.5 containing 3% (w/v) β‐CD—was equilibrated at 37.0 ± 0.5°C for 15 min. The donor solution was then removed, and the compartment was loaded with the film sample (0.95 cm^2^) and soaked with 200 μL of citrate buffer pH 5.5. The system was maintained at 37.0±0.5°C under constant magnetic stirring and protected from light for up to 3 h (end points: 30 min, 1 h, and 3 h). At predetermined time intervals, 500 µL aliquots of the receptor fluid were withdrawn and immediately replaced with an equal volume of fresh medium to maintain sink conditions within the system. The collected samples were frozen and subjected to freeze‐drying. The resulting dried residues were treated with 200 µL of an ethanol/methanol (1:1, v/v) mixture and left to stir at room temperature, protected from light, overnight. The samples were then centrifuged, and the supernatant was analyzed by HPLC–DAD, as previously described. Moreover, at the end of each experiment, the Franz cells were disassembled, and the buccal mucosa was washed, then subjected to hot methanolic extraction at 60.0 ± 0.5°C for 2 min. The extraction procedure was repeated twice, and the fractions collected were combined in a 5‐mL volumetric flask and brought to volume with methanol. The amounts of AG, QRC, and RSV extracted were determined by HPLC–DAD analysis, as previously described. Each experiment was performed in triplicate, and results are expressed as mean (*n* = 3) ± SE.

### 2.11 Data Analysis

The data are expressed as mean ± SE. All differences were statistically evaluated with Student’s *t*‐test or the one‐way analysis of variance (ANOVA or *F*‐test) with the minimum levels of significance with *p* < 0.05.

## 3 Results and Discussion

### 3.1 Multiple Maceration Procedure to Enhance Polyphenols Recovery from Waste Black Bentonite and Best Extract Selection

In recent years, the possibility of recovering polyphenols from BB, a waste material produced in large quantities by the grape processing and winemaking industries, has successfully emerged. This recovery was achieved through maceration performing green extraction with nonconventional solvents, with the aim of obtaining new secondary raw materials suitable for the development of cosmetics, medical devices, and pharmaceuticals. In our previous works, we reported the optimized maceration procedure in terms of: i) bentonite/solvent ratio, ii) time, iii) temperature, iv) best extraction solvent (PEG200). Moreover, the maceration process has been conducted by using both the BB wet (the actual recovered waste) and the BB dry (obtained following freeze‐drying of the BB wet) and considering the water content of the BB wet (55.32 ± 1.27%) to correct and maintain the bentonite/solvent ratio [24, 26, 27, 28]. In this work, the previously reported maceration procedure was repeated conducting a multiple maceration process consisting in seven consecutive cycles of maceration. This approach was aimed to obtain PEG200‐based extracts further enriched in polyphenols. To this end, a starting amount of the selected extraction solvent was sequentially treated with novel BB aliquots. The extracts obtained after each extraction cycle were characterized in terms of recovery %, density, pH, phenolic and protein contents, antioxidant power in order to identify the most cost‐effectiveness extract. The selected extract was then used for the development of buccal films suitable for the adjuvant treatment of oxidative stress‐related pathologies affecting the oral cavity (e.g., OLP, RAS, OM). To obtain a clearer understanding of the multiple maceration process, the procedure was carried out using both BB dry and BB wet. Furthermore, since the transition from a simple maceration to a multiple maceration procedure entails a scale‐up of the previously optimized extraction technique (from 12–200 mL of extraction solvent), the effect of different agitation mechanisms on the process was also investigated. Specifically:

• In the extractions indicated as DMa1‐7, “D” indicates that the extracted matrix was BB dry, “Ma” denotes that magnetic stirring was employed using a magnetic stir bar as previously described, and the numbers 1 to 7 refer to the extraction step.
• In the extractions denoted as WMa1‐7, “W” indicates that the extracted matrix was BB wet, “Ma” denotes that magnetic stirring was employed, and the numbers 1 to 7 again refer to the extraction step.
• In the extractions named as WMe1‐7, “W” again indicates that the extracted matrix was BB wet, while “Me” denotes that mechanical stirring was applied using a stirrer equipped with a Teflon blade of dimensions appropriate to the beaker employed. As before, the numbers 1 to 7 refer to the extraction step.

During the mixing phase, when using BB wet, it was immediately visually evident that the mechanical stirring promoted the formation of a better bentonite/solvent suspension, appearing smooth, homogeneous, and free from aggregates. In contrast, the simple magnetic stirring appeared insufficient when large volumes were employed, as the presence of bentonite aggregates was consistently observed at the end of the maceration process, possibly affecting the extraction itself. This issue did not occur in the DMa extraction series, likely due to the finer texture of the dry starting matrix. Preliminary investigations were conducted to evaluate the impact of the stirring technique also on the BB dry extract but both mechanical and magnetic stirring yielded comparable results. Furthermore, these results are consistent with those previously observed when considering both dry and wet white bentonite. For these reasons, the extraction carried out with BB dry was conducted only by magnetic stirring, as this approach is technically simpler to implement under laboratory conditions.

The first parameters that can be evaluated for each extraction step concern the recovery %, density, and pH after water dilution (Table 2). Regarding the recovery %, this refers to the amount of liquid extract recovered at each extraction step respect the initial liquid mass of PEG200. The recovery % values are particularly important in determining the feasibility and cost‐effectiveness of the process. Solvent loss mainly occurs because BB retains a certain amount of solvent even after prolonged centrifugation, remaining as a moist mass. Increasing the centrifugation speed or duration did not increase the recovery %. As reported, initially comparable recovery % values (e.g., ≈80% in the first step) among the DMa, WMa, and WMe extractions were obtained, while marked differences were observed in the final steps, with recoveries of 3.40%, 11.98%, and 8.20% for DMa‐7, WMa‐7, and WMe‐7, respectively. The higher recovery values obtained from extracts derived from BB wet, compared with those from BB dry, are likely associated with the presence of water in the starting waste. When mixed with PEG200, water increases the amount of recoverable liquid, thus apparently enhancing the overall recovery %. This effect, only slightly evident in the first extraction steps, becomes increasingly relevant in later steps, which involve successive additions of fresh portions of BB wet, each containing ≈55% of residual water. Consequently, the contribution of the water content becomes progressively more significant, resulting in marked differences in the solvent recovery % between extractions performed with BB wet versus BB dry. Considering that the density of PEG200 at 20°C is 1.115 ± 0.005 g/mL, a slight increase in native density was observed in all extracts. This increase was slightly more pronounced in the DMa series, probably because the progressive water retention occurring when using BB wet tended to counterbalance the overall density rise, making it less evident. The intrinsic color of the extracts, versus the native colorless extraction solvent, already proved that colored PEG200‐soluble compounds had been extracted from BB. This conclusion was further confirmed by measuring the pH after water dilution. The dilution of a PEG200 aliquot alone yielded a pH of 6.39, whereas the dilution of the extracts resulted in acidic pH values ranging from 3.2 to 4.0. This acidity is undoubtedly attributable to the presence of polyphenols and organic acids which are safe and naturally occurring in must and wine [34, 35]. A preliminary indicator of the progressive enrichment of the extraction solvent is, indeed, the step‐dependent decrease in pH.

**TABLE 2:** Extract characteristics: recovery % (*n* = 4), density (*n* = 12), and pH (*n* = 12). Mean ± SE.

Sample		Recovery %	Density, g/mL	pH
**DMa**	**DMa‐1**	76.36 ± 0.69	1.117 ± 0.010	3.92 ± 0.15
	**DMa‐2**	57.07 ± 1.08	1.132 ± 0.003	3.58 ± 0.15
	**DMa‐3**	41.50 ± 0.94	1.136 ± 0.015	3.66 ± 0.19
	**DMa‐4**	29.79 ± 0.97	1.149 ± 0.006	3.56 ± 0.11
	**DMa‐5**	18.71 ± 0.68	1.151 ± 0.006	3.52 ± 0.10
	**DMa‐6**	9.89 ± 0.93	1.163 ± 0.004	3.39 ± 0.03
	**DMa‐7**	3.40 ± 0.71	1.175 ± 0.007	3.35 ± 0.03
**WMa**	**WMa‐1**	79.84 ± 4.15	1.125 ± 0.013	4.00 ± 0.01
	**WMa‐2**	62.63 ± 3.08	1.113 ± 0.011	3.50 ± 0.04
	**WMa‐3**	45.11 ± 3.47	1.136 ± 0.012	3.32 ± 0.01
	**WMa‐4**	33.18 ± 4.16	1.136 ± 0.001	3.28 ± 0.02
	**WMa‐5**	26.88 ± 4.20	1.131 ± 0.005	3.25 ± 0.04
	**WMa‐6**	17.16 ± 4.66	1.137 ± 0.018	3.25 ± 0.01
	**WMa‐7**	11.98 ± 4.14	1.136 ± 0.009	3.21 ± 0.01
**WMe**	**WMe‐1**	78.94 ± 1.99	1.111 ± 0.011	3.69 ± 0.08
	**WMe‐2**	61.44 ± 2.99	1.134 ± 0.012	3.38 ± 0.04
	**WMe‐3**	42.63 ± 4.90	1.136 ± 0.011	3.35 ± 0.03
	**WMe‐4**	29.43 ± 6.08	1.127 ± 0.009	3.34 ± 0.04
	**WMe‐5**	16.59 ± 3.83	1.133 ± 0.005	3.36 ± 0.02
	**WMe‐6**	10.43 ± 4.36	1.142 ± 0.007	3.36 ± 0.01
	**WMe‐7**	8.20 ± 3.65	1.143 ± 0.009	3.37 ± 0.01

**FIGURE 1:**
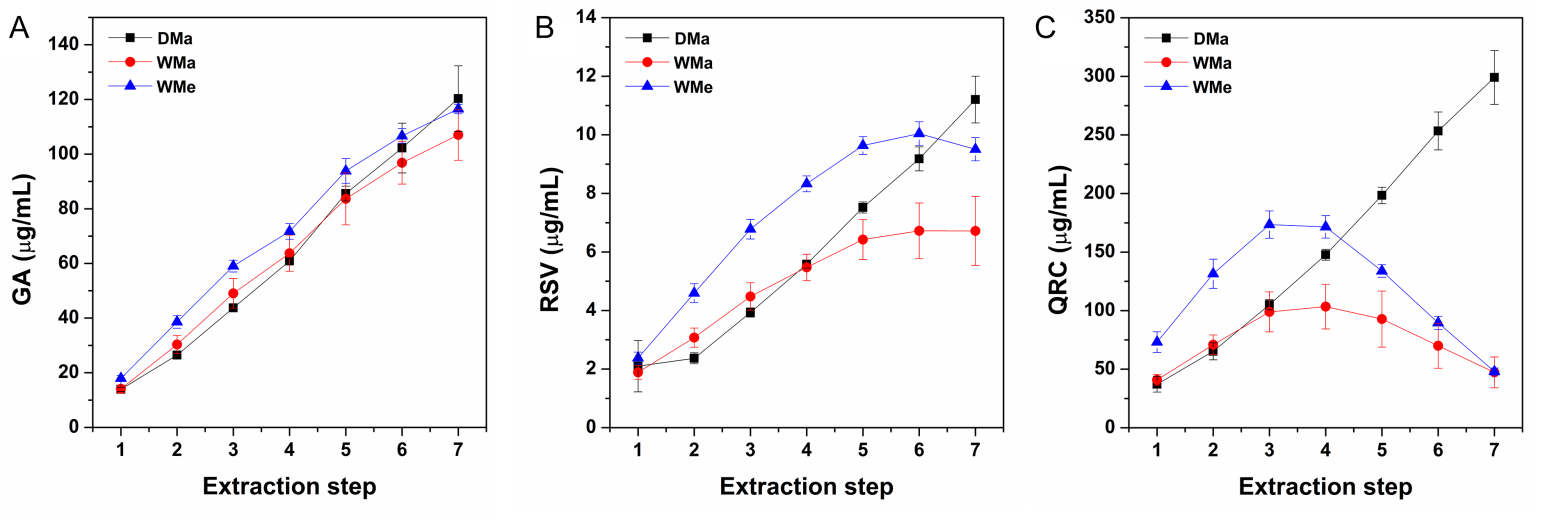
Amount of (A) GA, (B) RSV, and (C) QRC in the DMa, WMa, and WMe extracts as a function of the extraction step. Mean (μg/mL; *n* = 12) ± SE.

Further evidence supporting the previously proposed hypothesis (i.e., that the water fraction contained in the BB wet mixes with PEG200) was provided by the HPLC–DAD analyses, which enabled the quantification of GA, RSV, and QRC concentration in each extraction step (Figure 1). A distinctly different trend was immediately evident for the three polyphenols under investigation. The concentration of GA (Figure 1A) increased progressively in a step‐dependent manner across all samples. In contrast, RSV and QRC were extracted in a step‐dependent manner only in the DMa series, whereas a plateau was observed for RSV (Figure 1B), and even a progressive decrease in QRC concentration (Figure 1C) was recorded in samples obtained from BB wet. As theoretically an increasing trend, or at most a saturation trend leading to a plateau, would be expected, the obtained results could sound inconsistent. However, this apparent contradiction can be readily explained. Among the three compounds investigated, GA is the only reference molecule that is freely soluble in water and is therefore unaffected by the progressive introduction of water from the BB wet into PEG200 during successive extraction steps. In contrast, the situation changes markedly for QRC and RSV in all the extractions performed using BB wet. Both QRC and RSV are poorly water‐soluble compounds (QRC < RSV); therefore, the apparently anomalous behavior is not unexpected. On the contrary, it further supports the hypothesis that the addition of fresh aliquots of BB wet to the extraction liquor introduces additional amounts of water, which mix with PEG200. Initially, this mixture promotes the recovery of biophenols. However, as the number of extraction cycles increases, the contribution of water becomes sufficiently significant to hinder the solubility of QRC and RSV, leading not only to saturation phenomena (as observed for RSV), but also to a decrease in the extract amount of QRC. As the water content in the extraction liquor continues to increase, QRC progressively loses solubility, precipitates, and is readsorbed onto the bentonite surface, for which it evidently exhibits a greater affinity. The HPLC–DAD data also enabled other relevant considerations. Previous studies demonstrated a more efficient extraction from BB wet than from BB dry [28]; however, when observing the samples of the WMa series, this behavior was not reproduced, not even during the initial extraction steps. Moreover, the results obtained are in disagreement with the previous findings [24, 26, 27]. This discrepancy is probably related to the previously mentioned poor mixing of the BB wet with PEG200 when using a magnetic stir bar for large solvent volumes. This hypothesis is supported by the results obtained for the extracts of the WMe series. In particular, for RSV and QRC, the WMe series extracts exhibited higher content of each compound respect the DMa and WMa samples up to the sixth and fourth extraction steps, respectively, thus demonstrating greater extraction efficiency with fewer maceration cycles.

**FIGURE 2:**
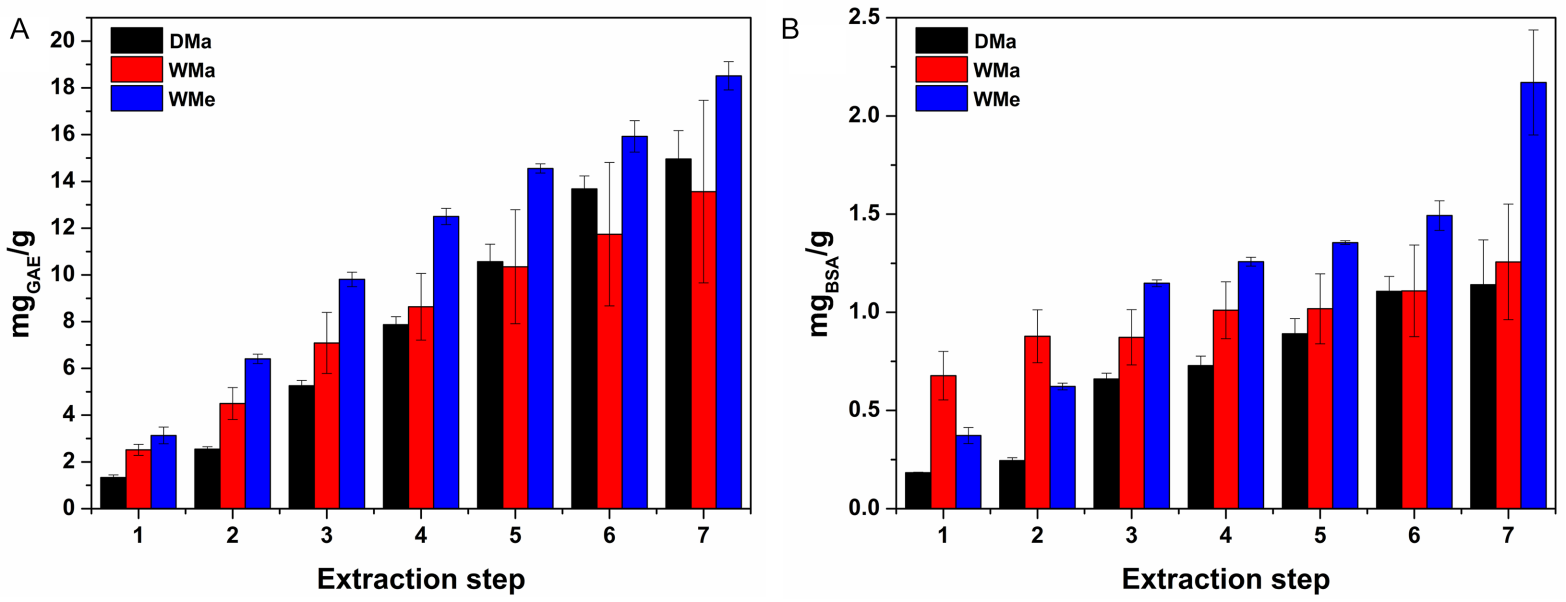
(A) TPC by the Folin–Ciocalteu assay expressed as mg_GAE_/g and (B) TPtC by the Bradford assay expressed as mg_BSA_/g in the DMa, WMa, and WMe extracts as a function of the extraction step. Mean (*n* = 12) ± SE.

The superiority of the WMe series was further confirmed by analyzing the total phenolic (Figure 2A) and protein (Figure 2B) contents as a function of the extraction steps. For all the extraction series, a step‐dependent increase in both TPC and TPCt was observed, and, overall, the WMe series appeared to be richer in both polyphenols and proteins at comparable extraction steps. It should also be emphasized that the primary aim of the here reported multiple maceration procedure was to enrich the extract in polyphenols, while the extraction of proteins occurred only as a secondary process. Nonetheless, the extraction procedure may be considered sufficiently selective, as the amount of proteins extracted is approximately one order of magnitude lower than that of phenols, despite the starting matrix being intrinsically richer in proteins than in phenolic compounds. This is because BB is used as a clarifying agent for the deproteinization of musts and wines, with the purpose of improving their organoleptic properties and long‐term stability. It is also worth noting that the proteins present in the starting matrix, and consequently in small quantities in the extracts, are entirely safe, as they are natural components of grapes. They are normally removed from musts and wines solely to meet consumers’ expectations and not because they pose any health risk [36].

**FIGURE 3:**
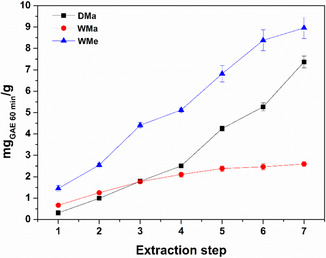
Antioxidant power of the DMa, WMa, and WMe extracts calculated by the DPPH assay and expressed in terms of mg_GAE_ _60 min_/g as a function of the extraction step. Mean (*n* = 12) ± SE.

The results obtained in terms of TPC were consistent with those observed in terms of antioxidant power, which was assessed through the DPPH assay and expressed as mg_GAE_ _60 min_/g (Figure 3). The superiority of the WMe series was immediately evident, showing an antioxidant activity that increased proportionally with the extraction step. Moreover, the trends observed for the DMa and WMa series were confirmed. The former, after the fifth extraction cycle, clearly outperformed the latter, although it remained significantly lower than the WMe series also in terms of scavenger properties.

Overall, it should be emphasized that the waste material derived from wine and must processing is the BB wet. Indeed, the clarification process involves the addition of BB to the product to be clarified, followed by stirring, sedimentation, and pressing, resulting in the separation of the clarified must/wine from the waste BB. Despite the pressing procedure, the BB remains wet and represents the waste material to be valorized. The corresponding BB dry was obtained in the laboratory through freezing and subsequent freeze‐drying of the BB wet in order to: i) determine the residual moisture content of the waste material; ii) evaluate its possible impact on polyphenols recovery; and iii) provide a comparison capable of supporting our hypotheses. As a consequence, proposing to further work with the BB dry would not be cost‐effective, as it would be unreasonable to couple a waste recovery process with an expensive and poorly scalable procedure such as freeze‐drying. Moreover, in view of potential future semi‐industrial and/or industrial scale‐up, particular attention should be devoted to the choice of the mixing method, since, as clearly demonstrated by the comparison between the WMa and WMe extractions, efficient mixing has a significant impact on the effectiveness of the maceration process. Finally, considering all the obtained results, the extract identified as the best compromise between high phenolic content, high antioxidant activity, and acceptable recovery % was the WMe‐3. This extract exhibited a recovery above 40%, representing the last extraction step that did not compromise QRC recovery, while displaying a TPC comparable to that of DMa‐5 and WMa‐5, and an antioxidant activity similar to DMa‐5. Therefore, this extract, hereafter recognized in this work as a new secondary raw material and designated as Bentophen200 extract, was selected for the development of buccal films with antioxidant properties, potentially useful to manage OM, OLP, and RAS lesions. Indeed, according to the literature, antioxidants exert a protective and pain relief effect against the mentioned oral lesions owing to their reactive oxygen species‐scavenging activity, regardless of the specific class of antioxidants employed. The literature is highly diverse in this regard and supports the use of various classes of compounds, including polyphenols. Generally, pure polyphenols are not directly employed; rather, plant extracts containing different classes of polyphenolic compounds are investigated, including flavonoids such as quercetin and curcuminoids such as curcumin [37, 38, 39, 40]. However, some specific polyphenols as gallic acid, epicatechin, apigenin, kaempferol, and quercetin emerged as antioxidant molecules with potential against OM [14]. As we previously conducted an in‐depth liquid chromatography–tandem mass spectrometry analysis of the polyphenolic footprint of our PEG‐based extracts, it should be highlighted that the extract composition includes the previously mentioned phenolic acids and flavonoids and could thus be considered potentially useful for the intended purpose [27].

### 3.2 Development and Characterization of Bentophen200 Extract‐Containing Buccal Films

An aspect that has received limited attention in the pathogenesis of OM, OLP, and RAS is the contribution of oxidative stress. This condition can cause damage to several cellular components, including proteins, membrane lipids, and nucleic acids. Moreover, the induction of possible genetic mutations may play a critical role in the potential malignant transformation of OLP lesions. Recent literature has demonstrated that antioxidants may not only effectively reduce pain, but also increase disease resolution rates and prevent both the potentially malignant outcomes of OLP and the onset of OM following chemo‐ or radiotherapy [14, 41]. In light of these considerations, Bentophen200 extract can be considered as a novel secondary raw material with potential utility for the development of formulations intended for the locoregional adjuvant treatment of the aforementioned pathologies. Among the various pharmaceutical dosage forms suitable for buccal administration, polymeric films are particularly noteworthy in this context. These are thin, flexible, and mucoadhesive preparations that are easy to self‐administer and generally patient‐friendly. In addition to acting as delivery systems for the incorporated active compounds, they can also protect the injured sites by exerting a wound‐dressing effect, which is beneficial in reducing patients’ nociceptive perception [42, 43, 44]. Among the techniques employed for the preparation of buccal films, the solvent casting method was selected in the present study. This method involves oven treatment of the aqueous dispersion containing the extract and the other component of the formulation. Solvent casting from water dispersions was chosen not only for its simplicity, but also to comply with a green formulation approach, as the use of water as the evaporating solvent is considered eco‐friendly. However, given the well‐documented thermal instability of polyphenols, it was necessary to preliminarily evaluate the stability of Bentophen200 extract under thermal treatment at 30 ± 1°C. Following 48 hr of thermal treatment, the loss of water from the extract was immediately quantified, resulting in a weight loss of 21.04 ± 0.11%. This substantial loss can be attributed both to processing losses (BB is an intrinsically moist matrix, as previously discussed) and to the multiple extraction procedure, leading to progressive water enrichment due to water–PEG miscibility. The results obtained from the characterization of the extract before and after oven treatment, corrected for the percentage of water loss, are shown in Table 3.

**TABLE 3:** Bentophen200 extract characterization: freshly prepared extract versus extract coming from the thermal treatment (oven, 30 ± 1°C, HR: 60%, 48 h). Mean (*n* = 3 for weight and density; *n* = 6 for the other parameters) ± SE.

Parameters		Bentophen200 extract, freshly prepared	Bentophen200 extract, thermal treatment
**Weight (g)**		2.11 ± 0.01	1.66 ± 0.01
**Density (g/mL)**		1.14 ± 0.01	1.15 ± 0.01
**HPLC–DAD**	GA (μg/mL)	66.27 ± 1.95	57.79 ± 3.89
	RSV (μg/mL)	7.74 ± 0.09	7.74 ± 0.16
	QRC (μg/mL)	195.46 ± 0.18	195.89 ± 3.03
**Folin–Ciocalteu**	CPT (mg_GAE_/g)	10.47 ± 0.08	10.90 ± 0.23
**DPPH assay**	mg_GAE_ _60 min_/g	4.86 ± 0.12	4.92 ± 0.08

**FIGURE 4:**
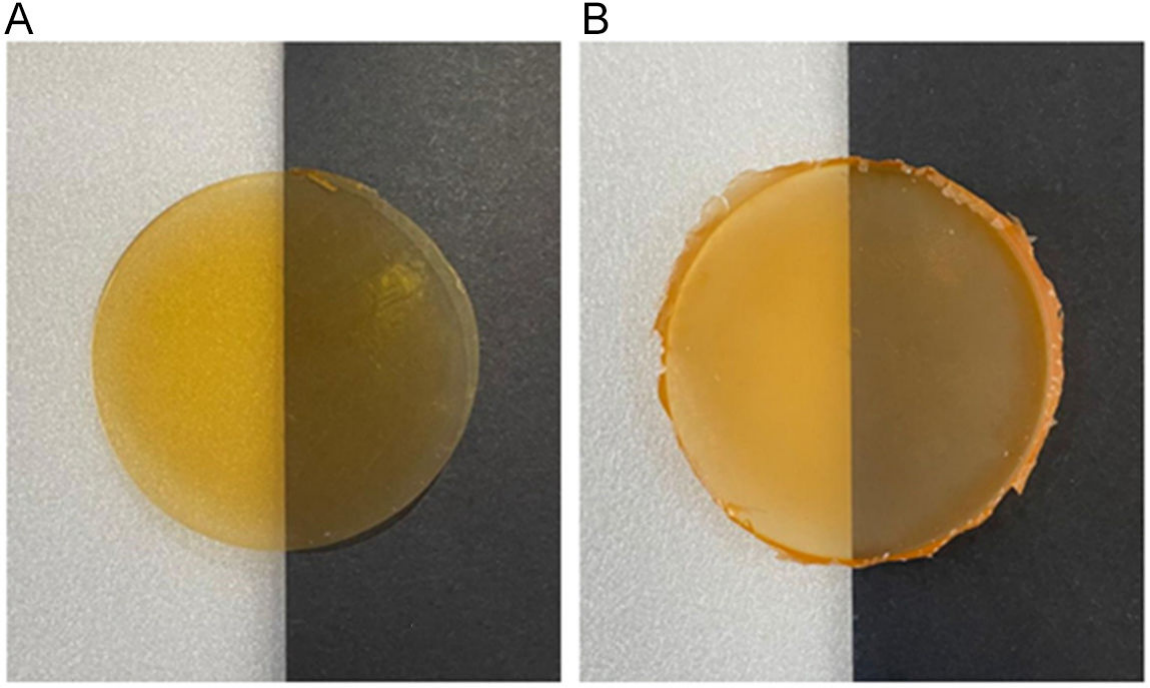
Appearance of the (A) BentoPect and (B) BentoPect‐Hyalu buccal films.

The results obtained demonstrate the stability of Bentophen200 extract under oven treatment, supporting its suitability for the formulation of mucoadhesive buccal films via the solvent casting technique under the explored parameters. This preliminary assessment confirmed that the mild thermal conditions required for solvent evaporation do not compromise the integrity of the extract, thereby validating the chosen manufacturing approach. Following compatibility screening with several natural, synthetic, and semisynthetic polymers, citrus pectin was identified as the most suitable film‐forming agent. Pectin combines biocompatibility, nontoxicity, edibility, and mucoadhesive properties, making it particularly appropriate for buccal administration [45]. In addition, its potential derivation from agrifood waste further enhances the sustainability profile of the formulation [46, 47, 48]. Furthermore, recent studies have shown that pectin and polyphenols are capable of forming complexes. Such interactions represent a rapidly evolving research frontier across several disciplines, including food science, cosmetics, materials science, and engineering [49]. The ability of pectin to form crosslinked networks in the presence of calcium ions represents a key advantage for film formation. The calcium‐to‐pectin ratio is a critical parameter. Although calcium chloride is commonly reported as the preferred crosslinking agent, calcium lactate pentahydrate was selected due to its potential superior performance for the intended purpose. Indeed, while Ca^2+^ ions could ensure effective crosslinking between pectin chains, the hygroscopic lactate counterion could contribute to improve film flexibility by retaining water molecules within the gel network. This effect was further supported by the presence of PEG200, the main component of Bentophen200 extract, which is widely recognized for its plasticizing properties [50]. To further enhance the therapeutic potential of the films, hybrid polymeric systems were developed by incorporating hyaluronic acid (HA), owing to its well‐established wound‐healing properties. Polymeric dispersions at 3% (w/w) of pectin and 1% (w/w) of HA were identified as optimal, providing an appropriate balance between processability and drying efficiency during solvent casting. Additionally, the choice of mold material significantly influenced film quality. Silicone substrates yielded inhomogeneous films with visible air entrapment, whereas polystyrene molds enabled the production of visually uniform, defect‐free films and were therefore selected. Overall, formulation studies led to the optimization of two buccal film systems: BentoPect (Figure 4A), based on Bentophen200 extract, pectin, and calcium lactate; and BentoPect‐Hyalu (Figure 4B), additionally containing hyaluronic acid. Both films exhibited an orange coloration attributable to the presence of the colored Bentophen200 extract. The BentoPect film is transparent, whereas the BentoPect‐Hyalu appeared opaque, a feature already evident during preparation of the polymeric dispersion and attributable to the presence of HA. This behavior is likely due to interactions between HA and Ca^2+^ ions in the dispersion, which, as reported in the literature, induce opalescence in HA‐based films in a concentration‐dependent manner [51].

**FIGURE 5:**
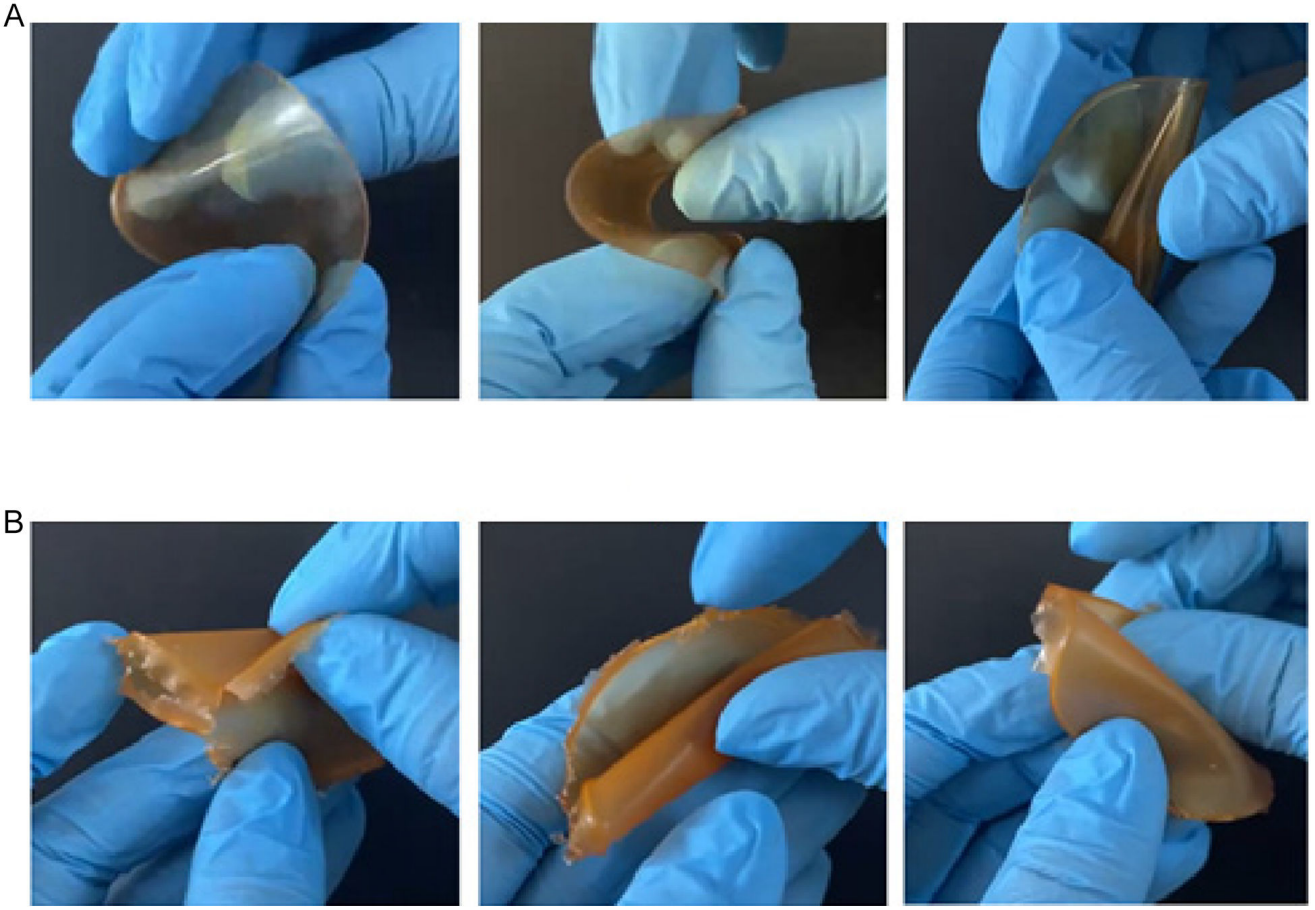
Flexibility evaluation of the (A) BentoPect and (B) BentoPect‐Hyalu buccal films.

These formulations were then immediately evaluated in terms of flexibility as this is a crucial feature of effective buccal films which might move according to the normal oral cavity movement without breaking. In terms of folding endurance, both films remained intact after 300 folding cycles and were then considered resistant and flexible. Their behavior is observable also through the reported photographs (Figure 5).

These formulations were subsequently characterized to evaluate their physicochemical and functional performance. First, the dry films were weighted and, as reported in Table 4, notwithstanding experimental error, the final weight of the two formulations was the same. Moreover, the low SE values indicated the reproducibility of the preparation process. The even low residual water content could be attributed to the presence of hygroscopic components such as pectin, hyaluronic acid and, in particular, the lactate ion. Moreover, one of the most significant advantages in the administration of buccal films relies in the possibility of tailoring drug delivery. Indeed, variable size film portions could be cut, allowing the modulation of the amount of actives administered at the site of application. However, this versatility dependents on the uniformity of the final product. As shown in Table 4, both buccal films resulted homogeneous in terms of weight, thickness, and actives content, as suggested by the limited SE values. Some further aspects require particular attention, First, it is important that buccal films are thin in order to avoid discomfort to the patient once applied. Additionally, it is relevant to mention the method used to evaluate the LE%. Since the Bentophen200 extract contains a rich and heterogeneous polyphenolic pool, the quantification of all the actives present in the extract is impractical. Therefore, this evaluation was carried out in terms of TPC and antioxidant activity which, normalized to the values obtained for the extract, led to the LE%.

**TABLE 4:** Uniformity study of the BentoPect and BentoPect‐Hyalu buccal films in terms of weight (*n* = 12), thickness (*n* = 15), LE%, CPT, and antioxidant power (*n* = 6). Mean ± SE.

	BentoPect	BentoPect‐Hyalu
**Weight of the film (g)**	1.04 ± 0.01	1.08 ± 0.04
**Residual water content %**	0.77 ± 0.07%	1.27 ± 0.29%
**Weight per disk (0.38 cm^2^) (mg)**	14.56 ± 0.23	13.63 ± 0.35
**Thickness (mm)**	0.39 ± 0.01	0.39 ± 0.01
**LE%**	98.12 ± 1.45	98.67 ± 1.73
**CPT (mg**_**GAE**_ **/cm^2^)**	0.484 ± 0.007	0.487 ± 0.009
**Antioxidant power (mg**_**GAE**_ _**60 min**_ **/cm^2^)**	0.028 ± 0.001	0.027 ± 0.001

Further relevant parameters to be evaluated when dealing with buccal films are the swelling index and the mucoadhesive properties of the formulations.

**FIGURE 6:**
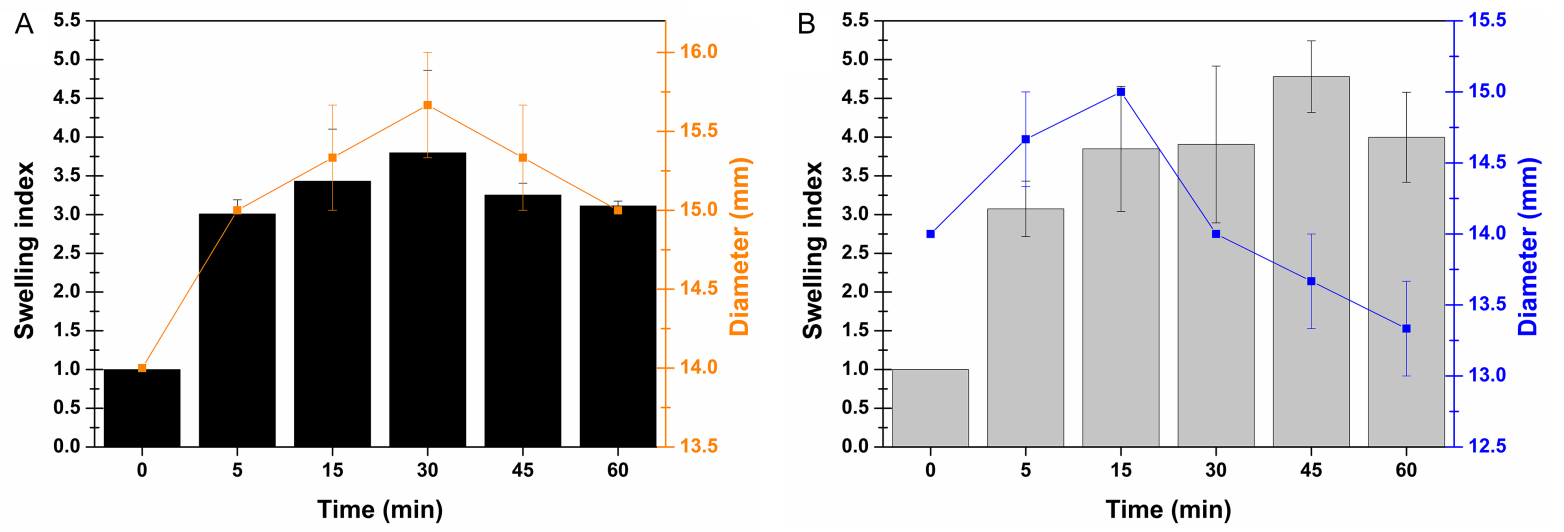
Swelling index and film disk diameter as a function of time when evaluating the (A) BentoPect and (B) BentoPect‐Hyalu buccal films. Mean (*n* = 3) ± SE.

The swelling index is a key parameter as mucoadhesion, patient compliance, and actives release are phenomena closely related to it. Indeed, excessive swelling of the formulation could favor its detachment from the site of application, and the patient may experience discomfort if the formulation, upon swelling, became thicker. Otherwise, swelling of the dosage form influences the release of the active substances from the dried polymeric matrix and, consequently, the effectiveness of the drug delivery process. The swelling test was performed by evaluating both the increase in weight of a portion of the film over time and the variation in its diameter and thickness. As shown in Figure 6, both films immediately absorb large amounts of water, up to approximately quadrupling their weight in the case of the BentoPect formulation and quintupling it for BentoPect‐Hyalu after 30 and 45 min, respectively. This behavior can be attributed to the nature of the films which are mainly composed by hygroscopic materials. The experimental data also evidenced that the presence of HA increases the swelling of the formulation. This effect could be attributed both to a greater spacing between the meshes of the crosslinked pectin network due to the presence of HA as well as to a possible interaction between Ca^2+^ ions and HA, which may result in a lower degree of pectin crosslinking. The swelling results obtained in terms of weight evaluation may appear to be inconsistent with those shown in terms of diameter variation. In this case, the BentoPect films increase their diameter up to 30 min, after which a reduction is observed. A markedly different trend is instead observed for the BentoPect‐Hyalu film, which was characterized by an increase in diameter during the first 15 min, followed by a clearly decreasing trend. In both cases, the reduction in diameter can be attributed to erosion and dissolution phenomena. Specifically, in the case of the BentoPect‐Hyalu, the presence of HA may promote a greater water uptake, as observed in terms of weight variation, thereby accelerating the solubilization/dispersion of the formulation components compared with the BentoPect film. This explanation is also visually supported by Figure S1 (see Supporting Information (SI)). The BentoPect disk swelled while maintaining its physical integrity, whereas the BentoPect‐Hyalu one swelled but also began to dissolve. The morphological variations of the films were also evaluated in terms of thickness. Yet, as shown in Figure S1 (see SI) no relevant variations in thickness were observed for either film, which thus potentially remain comfortable for patients.

**FIGURE 7:**
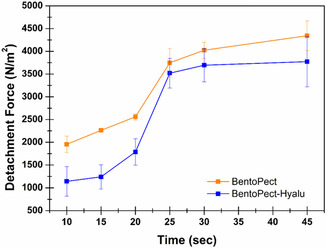
Detachment force (N/m^2^) as a function of time for the BentoPect (orange) and BentoPect‐Hyalu buccal films. Mean (*n* = 6) ± SE.

Mucoadhesion is essential to ensure the effectiveness of an oromucosal formulation. A mucoadhesive film ensures prolonged residence of the active compounds at the site of administration, providing intimate contact with the tissue where the lesion is located, preventing swallowing of the formulation, and promoting both patient compliance and adherence to the treatment. Then, both qualitative and quantitative ex vivo mucoadhesion studies were performed. The qualitative studies consisted in the application of film disks onto porcine buccal tissue pretreated with artificial saliva at body temperature. The complete adhesion of the disks to the buccal surface is shown in Figure S2 (see SI). Resistance to mechanical “stress” was evaluated by rotational movements, inversion, and bending. In addition, the ability of the formulation to adapt to tissue movements should be highlighted, in agreement with the results obtained in terms of flexibility. The quantitative studies were conducted by tensile strength test, using a Texture Analyzer. The obtained force of adhesion (N) values were then converted into detachment force (N/cm^2^) values by normalizing the force of adhesion to the contact surface area. Results are reported as a function of preload time (sec) (Figure 7).

As observable, for very short contact times (10–20 sec) the BentoPect films appear to be more mucoadhesive than the BentoPect‐Hyalu films, whereas for longer contact times (≥25 sec) the observed differences were not statistically significant. The difference observed in the initial phase of the mucoadhesion curves may be correlated with the presence of HA, a polymer that is well known to be polyanionic. Pectin, which is present in both formulations, also exhibits polyanionic characteristics; however, part of its negative charges is involved in crosslinking with Ca^2+^ ions. The presence of HA, on the other hand, introduces additional negative charges that may initially hinder contact with the mucosal tissue, which is itself negatively charged, due to electrostatic repulsion. This phenomenon is subsequently mitigated, as it is well known that the interactions responsible for mucoadhesion are multiple (e.g., hydrogen bonding and van der Waals interactions) [52, 53]. Overall, the results obtained are highly positive for both formulations, which demonstrate clear mucoadhesive properties. The literature does not report well‐defined threshold values for defining mucoadhesive formulations; however, detachment force values between 200 and 1000 N/m^2^ generally indicate moderate mucoadhesion [31], whereas values above 1000 N/m^2^ are indicative of good mucoadhesion [54, 55]. The films proposed here exhibit detachment force values of ≈3500 N/m^2^ for preload time above 25 sec. It is relevant to emphasize that the preload time can provide information regarding the duration for which pressure must be applied to the formulation, while placing it in contact with the mucosa, in order to allow the establishment of a stable mucoadhesive interaction. It therefore has a significant practical impact, as the formulations under investigation are intended for application to injured and painful sites within the oral cavity. Consequently, the requirement for short preload times would certainly result in greater patient compliance.

**FIGURE 8:**
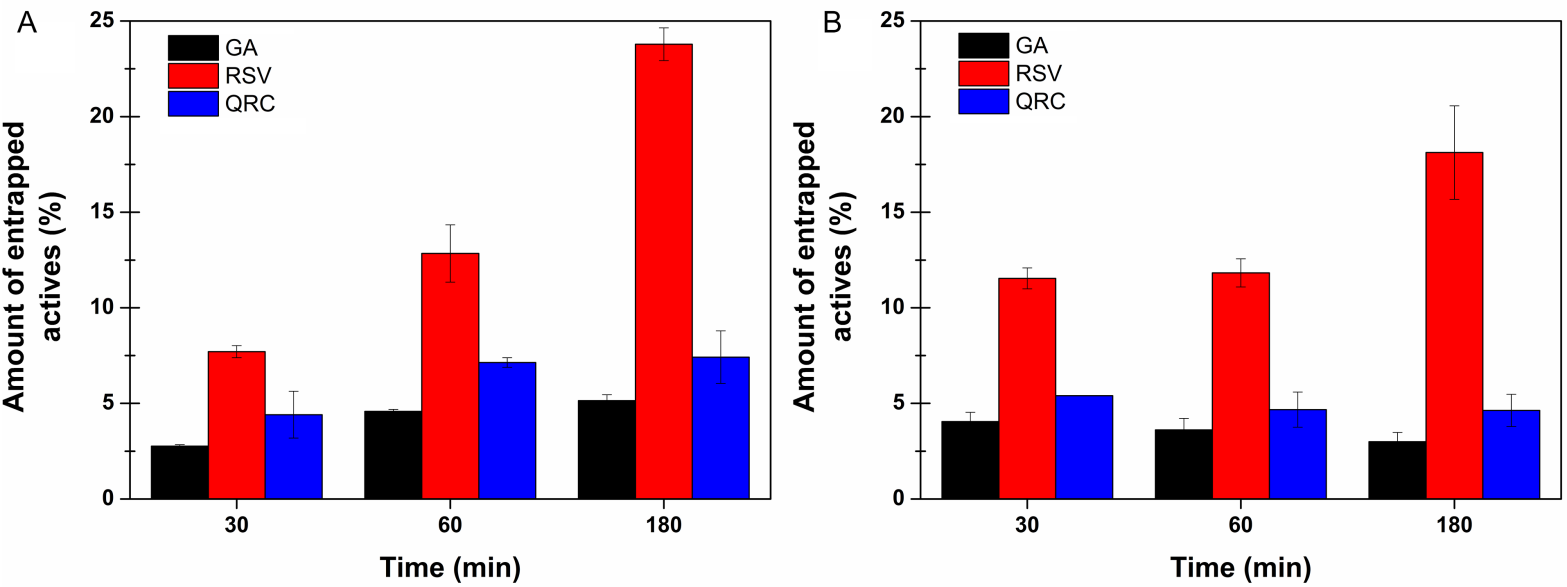
Percentage among of GA, RSV, and QRC found into the buccal mucosa at the end of the ex vivo permeation study as a function of time following the application of (A) BentoPect and (B) BentoPect‐Hyalu buccal films. Mean (*n* = 3) ± SE.

Finally, to evaluate the ability of the formulations to promote the interaction between polyphenols and oral tissues, as it is directly related to their potential usefulness, ex vivo permeation studies were conducted. For this purpose, vertical Franz diffusion cells were used as a well‐established two‐compartment open model, and porcine buccal mucosa was employed as a tissue highly similar to human mucosa. To avoid misleading results, both the donor and acceptor compartments were filled with citrate buffer pH 5.5, which is known to stabilize polyphenols [29, 56, 57]. Additionally, β‐cyclodextrins were included in the acceptor fluid, as they are able to increase the solubility of polyphenols in aqueous media [29, 58], thereby ensuring the maintenance of sink conditions. Again, as it was not possible to evaluate all the component of the Bentophen200 extract, the experimental results were focused on the quantification of GA, RSV, and QRC, previously mentioned as reference polyphenols for the obtained extract. As a result, the mentioned polyphenols were not able to permeate the mucosa. After 3 hr of experiment, HPLC–DAD analysis revealed no detectable presence of GA, RSV, or QRC in the acceptor compartment. Furthermore, the obtained chromatograms were essentially flat, indicating the absence not only of these three compounds but also of other constituents of the pool contained within the Bentophen200 extract. Concurrently, the ability of the reference polyphenols to accumulate within the buccal mucosa was assessed through extraction from the tissue following different contact times with the formulations. As shown in Figure 8, a time‐dependent accumulation behavior is generally observed, which is particularly pronounced for RSV. After 3 hr, the 23.79 ± 0.09% and 18.12 ± 2.45% of the RSV dose loaded within the formulation were found in the tissue following the application of BentoPect and BentoPect‐Hyalu, respectively. Interestingly, when comparing polyphenols accumulation over time, the BentoPect‐Hyalu film appeared superior at shorter times (e.g., 30 min), whereas an inversion is observed at 180 min.

This is presumably due to the pronounced swelling behavior of BentoPect‐Hyalu, which promotes more rapid release of the active compounds from the film than the BentoPect formulation. However, as already reported, this characteristic did not confer a long‐term advantage, as the film subsequently eroded and dissolved, losing the ability to maintain close and stable contact between the actives and the target tissue. Therefore, BentoPect promoted higher mucosal accumulation of polyphenols than BentoPect‐Hyalu. The prolonged dissolution time of the BentoPect films was further confirmed during the disassembly of the diffusion cells. Following 3 hr of experiment, the BentoPect film disk was swollen and occupied the entire available exchange surface yet remaining intact, whereas the BentoPect‐Hyalu film disk was completely dissolved.

## 4 Conclusions

This study introduces a new mucoadhesive buccal films dubbed “BentoPect” consisting of citrus pectin embedded with biophenols sustainably sourced from waste black bentonite using polyethylene glycol 200 as green solvent. The green approach of the research is reflected in three key aspects: i) recovery and valorization of a waste material through a multiple extraction process aimed at maximizing polyphenol recovery while identifying the compromise between phenolic content and extract recovery; ii) environmentally friendly film production by solvent casting from aqueous solutions; iii) selection of pectin as the main polymeric component of the film, as this biopolymer can also be obtained through waste recovery processes.

In view of potential semi‐industrial or industrial scale‐up, agitation of the bentonite/PEG200 suspension is a crucial parameter for optimizing polyphenol extraction, as demonstrated by the comparison between WMa and WMe extraction series. Among the obtained extracts, WMe‐3 provided the best compromise between high phenolic content, strong antioxidant activity, and acceptable recovery % (>40%), without significantly affecting QRC and RSV recovery from the water‐containing BB extraction matrix. Consequently, WMe‐3 was selected as the new antioxidant secondary raw material (Bentophen200 extract) for the development of buccal films useful for the prevention and treatment of oxidative stress‐related oromucosal pathologies.

Bentophen200 was proven to be a versatile raw material, stable following treatment at 30°C, and thus suitable for the preparation of polymeric films by solvent casting. The use of water‐dispersible polymers (e.g., hyaluronic acid and/or pectin) enabled water to be employed as the removable solvent, while citrus pectin was chosen due to its ability to stabilize polyphenols. Both BentoPect and BentoPect‐Hyalu films were highly reproducible, homogeneous, flexible, and mucoadhesive. However, BentoPect showed greater mucosal accumulation of polyphenols than BentoPect‐Hyalu. Its longer disintegration time, stronger mucoadhesion at very short preload times and lower swelling degree, identified BentoPect as the most suitable formulation. Overall, BentoPect film appears capable of promoting prolonged intimate contact with the mucosa and might then provide improved sustained release, wound‐dressing action, and pain relief.

## Funding

This work was supported by Ministero dell'Università e della Ricerca, Regione Siciliana, Università degli Studi di Palermo (Grants FFR‐D15‐008633, FFR‐D15‐502204).

## Conflicts of Interest

The authors declare no conflicts of interest.

## Supporting information

Supplementary Material

## Data Availability

The data that support the findings of this study are available from the corresponding author upon reasonable request.
