# Supplementary material for: High Value‐Added Secondary Raw Material From Winemaking Waste Bentonite to Design Antioxidant Pectin‐Based Mucoadhesive Buccal Films
Source: ChemistryOpen. 2026 May 29;15(6):e70236. doi: 10.1002/open.70236 (PMC13239696; doi:10.1002/open.70236)
Supplement: Supplementary file 1 — Supplementary Material [file OPEN-15-e70236-s001.pdf]

Supplementary materials

High value-added secondary raw material from winemaking waste bentonite to design antioxidant pectin-based mucoadhesive buccal films

Giulia Di Prima*[a], Cecilia La Mantia[b], Viviana De Caro[a,c]

[a] Dr. G. Di Prima, PhD; Prof. V. De Caro, PhD
Dipartimento di Scienze e Tecnologie Biologiche, Chimiche e Farmaceutiche (STEBICEF)
University of Palermo
Via Archirafi 32, 90123, Palermo, Italy
E-mail: giulia.diprima@unipa.it

[b] Dr. C. La Mantia
Dipartimento di Medicina di Precisione in Area Medica, Chirurgica e Critica (MePreCC)
University of Palermo
Via Liborio Giuffè 5, 90127, Palermo, Italy

[c] Prof. V. De Caro, PhD
Centro interdipartimentale di Riutilizzo bio-based degli scarti da matrici agroalimentari (RIVIVE)
University of Palermo
Viale delle Scienze, Ed. 14, 90128, Palermo, Italy

Data reported as supplementary materials:

- **Figure 1S:** Visual evaluation of the swelling degree of BentoPect and BentoPect-Hyalu buccal films at different time points in terms of diameter and thickness;
- **Figure 2S:** Ex vivo qualitative mucoadhesion test of BentoPect and BentoPect-Hyalu buccal films: evaluation of their ability to adhere to the mucosa and to remain attached even after rotational stress

WILEY-VCH

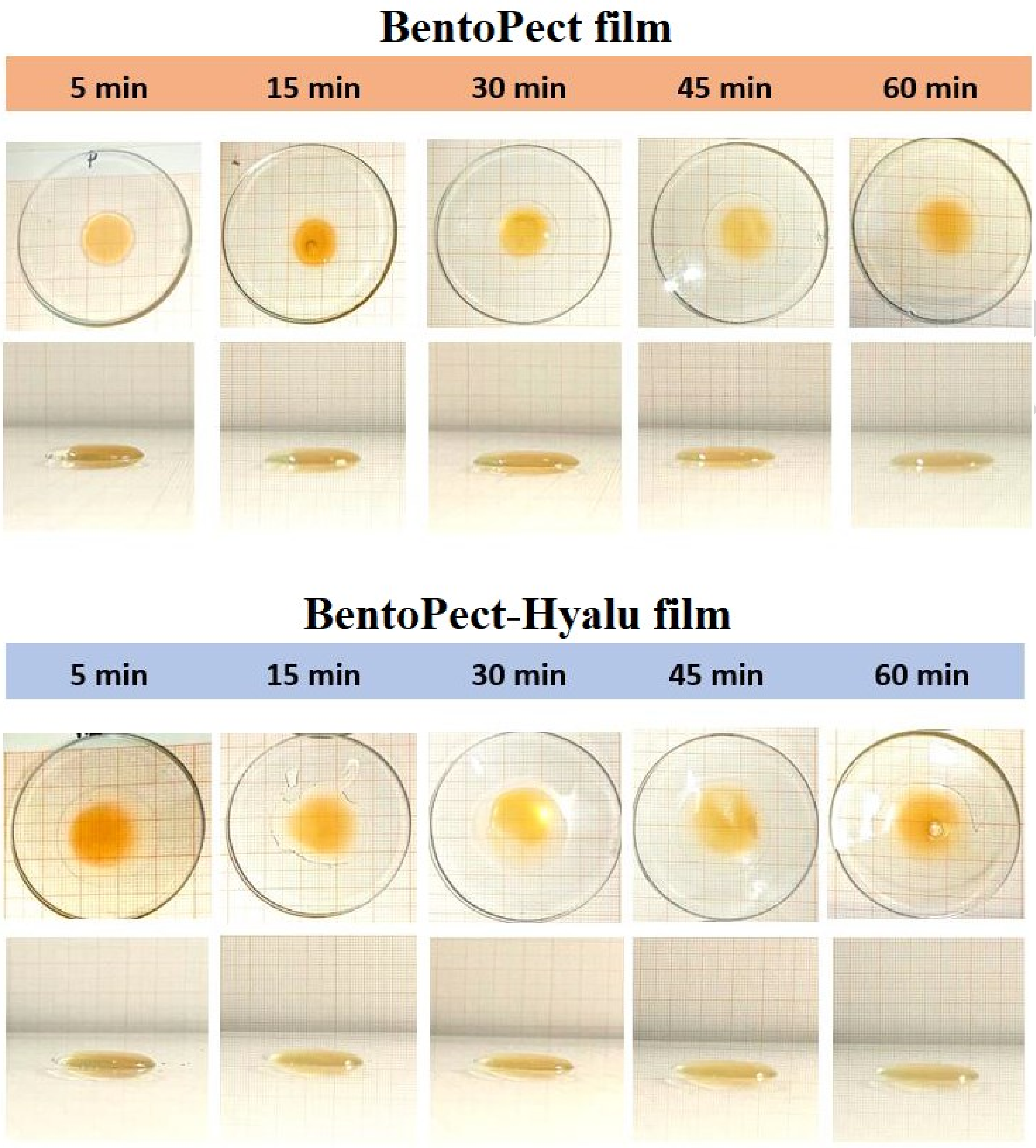

Figure 1S. Visual evaluation of the swelling degree of BentoPect and BentoPect-Hyalu buccal films at different time points in terms of diameter and thickness

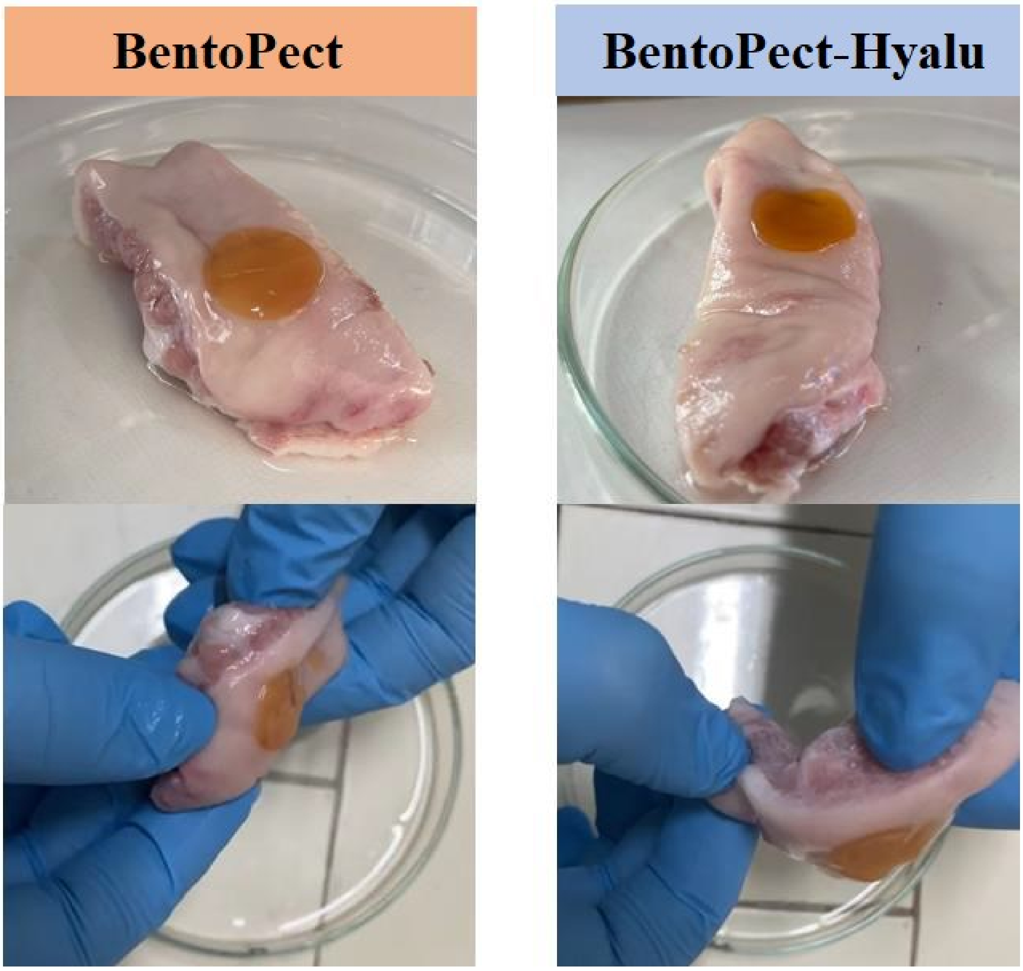

Figure 2S. Ex vivo qualitative mucoadhesion test of BentoPect and BentoPect-Hyalu buccal films: evaluation of their ability to adhere to the mucosa and to remain attached even after rotational stress

WILEY-VCH
